# Giant Renal Angiomyolipoma without Fat Density on CT Scan: Case Report and Review of the Literature

**DOI:** 10.1100/tsw.2010.135

**Published:** 2010-07-07

**Authors:** Kenneth G. Nepple, Nathan A. Bockholt, Laila Dahmoush, Richard D. Williams

**Affiliations:** Departments of Pathology and Urology, University of Iowa, Iowa City, IA, USA

**Keywords:** renal mass, kidney neoplasms, angiomyolipoma, computed tomography, nephrectomy

## Abstract

Giant renal angiomyolipomas have been reported, but typically have the pathognomonic finding of fat density on CT scan. We present the case of a 53-year-old male with a symptomatic, 35-cm, predominantly cystic renal mass without fat density on CT that on nephrectomy was found to be a fat-poor angiomyolipoma with predominantly epithelioid morphology weighing 17.9 kg. Giant renal angiomyolipoma without macroscopic fat density on CT scan is an exceedingly rare occurrence.

## INTRODUCTION

Large, solid, enhancing renal masses are most frequently renal cell carcinoma (RCC), but can be less common entities, including angiomyolipoma (AML), oncocytoma, urothelial carcinoma, sarcoma, or metastatic tumor. We present the unusual case of a giant cystic renal mass suspected to be RCC that was subsequently discovered to be fat-poor AML with predominantly epithelioid morphology.

## CASE PRESENTATION AND MANAGEMENT

A 53-year-old male had progressive weight gain over a 2-year period, from 240 to 310 lbs, with increasing abdominal girth. He then developed early satiety with postprandial nausea and emesis. CT scan with contrast demonstrated a primarily cystic right renal mass, 35 cm in greatest dimension, with a thin rim of enhancing tissue around the margins of the mass (Fig. 1). No fat density was present in the renal mass on CT scan. The renal vein, vena cava, and adrenal gland were displaced from the renal mass (Fig. 2), but otherwise normal. The renal mass was suspected to be cystic RCC. A renal biopsy was not performed due to the cystic nature of the mass. Metastatic evaluation was negative, including chest CT, bone scan, and brain MRI.

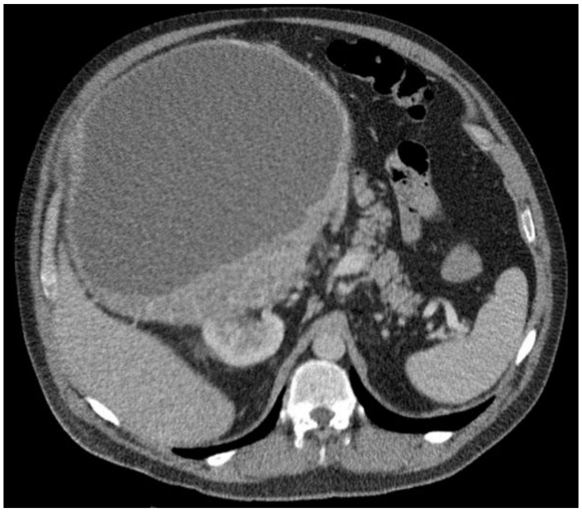

**FIGURE 1.** Transaxial CT scan image of large right renal cystic mass with enhancing peripheral rim.

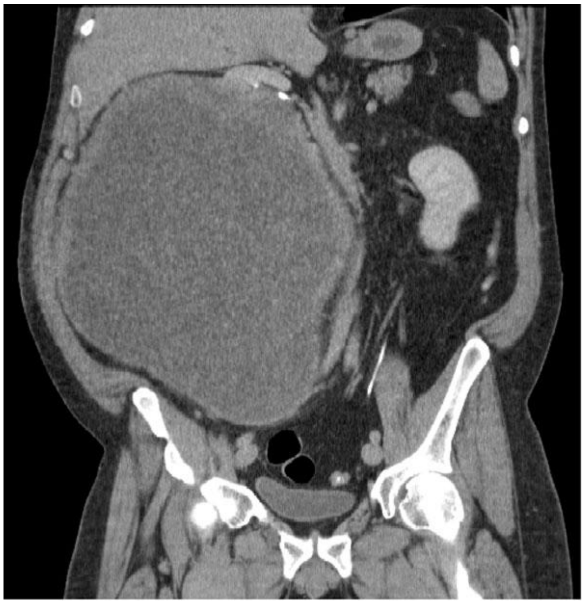

**FIGURE 2.** Coronal CT scan image of large right renal mass showing displacement of other abdominal structures.

Surgical treatment was recommended, and the patient underwent right open radical nephrectomy with lymphadenectomy through a bilateral subcostal (Chevron) incision. The renal mass was able to be separated from the surrounding structures with no direct extension (Fig. 3), with an estimated blood loss of 700 mL. Surgical specimen was a right kidney that weighed 17.9 kg with a 35-cm, in greatest dimension, cystic mass. On gross pathologic examination, the renal mass was primarily composed of necrotic cyst fluid with a minor component of solid tissue. Final pathology was AML with predominantly epithelioid morphology, which on immunohistochemistry stained positive for HMP-45 (Fig. 4), a monoclonal antibody against melanoma-associated antigen that differentiates AML from sarcoma[1]. All surgical margins and lymph nodes were negative. The patient had no evidence of recurrence and normal creatinine at follow-up 5 months postoperatively.

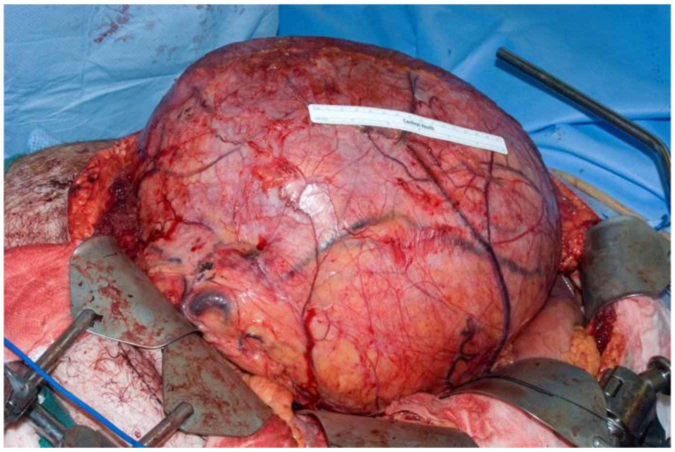

**FIGURE 3.** Intraoperative image during right nephrectomy.

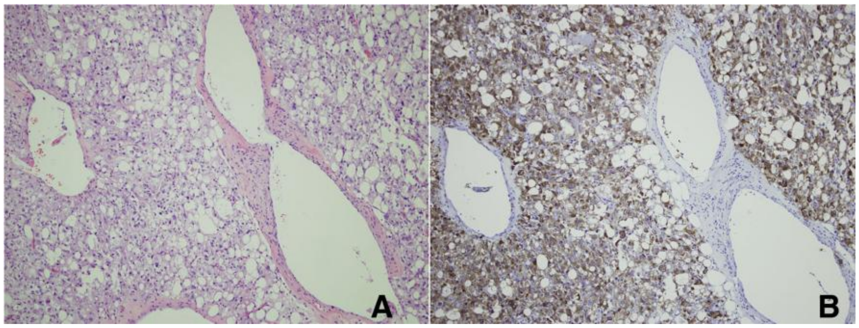

**FIGURE 4.** Microscopic image of AML with predominantly epithelioid morphology (A) with positive staining for HMB-45 (B).

## COMMENT

An AML is a benign mass consisting of blood vessels, smooth muscle, and adipose tissue components that can be seen in association with tuberous sclerosis complex; however, 80% of AMLs are not complex associated[2]. AML can be diagnosed on CT imaging based on the pathognomonic finding of even a small amount of fat (low attenuation of negative 20 or lower Hounsfield units) within a renal mass that virtually excludes the diagnosis of RCC[3,4]. The use of thin sections, noncontrast imaging, or pixel mapping may help to improve the sensitivity of CT in detecting AML[5].

The AML in this case had no fat density on CT scan, which had been reported in approximately 15% of all AMLs[6]; however, two recent series reported that 65–73% of surgically resected renal masses proven to be AML histologically, specifically fat-poor AMLs, were not suspected radiographically[7,8]. The use of MRI and ultrasound have been shown to be ineffective at identifying fat-poor AML when not suggested by CT findings[8]. The lack of radiographic fat in the current case can be explained by the combination of a cystic mass and a predominantly epithelioid histologic morphology. The epithelioid variant of AMLs may not have visible radiographic fat density because tumors are composed predominantly of epithelioid smooth muscle cells with minimal adipose tissue[9], and one institutional review of surgically resected AMLs reported epithelioid histology in 8% of cases, while 76% had classic triphasic histology[7]. The epithelioid histology variant of AML has been associated in rare cases with malignant and metastatic potential[10,11], although the vast majority are benign[7]. The epithelioid variant is relatively more common than its histologic counterparts in larger renal masses and tuberous sclerosis[7,12].

The 35-cm size of the AML in this case is unusually large. Our patient was an obese man weighing 240 pounds at baseline, and his sizable abdominal girth may have allowed the renal mass to grow to a remarkable size before prompting him to seek evaluation. The Cleveland Clinic group reported 209 AMLs surgically treated over 26 years with a median size of 4 cm and 75% of masses were ≤8 cm[7]. Another report of 60 patients with AML diagnosed over 16 years reported a median size of 4 cm, with the largest measuring 40 cm[13]. Several authors have used the term "giant" or "massive" AML to describe large AMLs. Case reports of AMLs reported as giant AMLs typically are greater than 15 cm[14,15,16,17,18,19], ranging up to 35 cm[20,21] and 45 cm[22] in size.

Giant AMLs without any radiographic fat density on CT scan have been reported in rare cases. Yamamoto et al. reported a 20-cm renal mass with multiple metastatic sites in lymph nodes, liver, lung, and spine that was found (postmortem) to be malignant epithelioid AML, which on previous CT scan did not have fat density[10]. To our knowledge, the current 35-cm renal mass is the largest AML ever reported without fat density present on CT scan, and its epithelioid histology, right-sided location, and male gender make it even more remarkable.

Management of AML is based on the risk of hemorrhage in masses with radiographic fat present and on the concern for malignancy in those masses without fat density on CT scan. Small renal AMLs <4 cm have traditionally been managed conservatively based on their decreased propensity for hemorrhage, while masses >4 cm can be effectively treated with selective embolization rather than partial or radical nephrectomy[23,24]. In rare cases, giant AMLs are followed conservatively to avoid hemodialysis, but those patients continue to be at risk of bleeding episodes[17]. The renal mass in our case clearly warranted nephrectomy due to the concern for possible RCC.
